# Pushing the limits of remote RF sensing by reading lips under the face mask

**DOI:** 10.1038/s41467-022-32231-1

**Published:** 2022-09-07

**Authors:** Hira Hameed, Muhammad Usman, Ahsen Tahir, Amir Hussain, Hasan Abbas, Tie Jun Cui, Muhammad Ali Imran, Qammer H. Abbasi

**Affiliations:** 1 University of Glasgow, James Watt School of Engineering, Glasgow, G12 8QQ UK; 2 School of Computing, Engineering and Built Environment, Glasgow Caledonian University, Glasgow, G4 0BA UK; 3 Department of Electrical Engineering, University of Engineering and Technology, Lahore, Pakistan; 4 School of computing, Edinburgh Napier University, Scotland, UK; 5 State Key Laboratory of Millimetre Waves, Southeast University, Nanjing, China

**Keywords:** Electrical and electronic engineering, Quality of life

## Abstract

The problem of Lip-reading has become an important research challenge in recent years. The goal is to recognise speech from lip movements. Most of the Lip-reading technologies developed so far are camera-based, which require video recording of the target. However, these technologies have well-known limitations of occlusion and ambient lighting with serious privacy concerns. Furthermore, vision-based technologies are not useful for multi-modal hearing aids in the coronavirus (COVID-19) environment, where face masks have become a norm. This paper aims to solve the fundamental limitations of camera-based systems by proposing a radio frequency (RF) based Lip-reading framework, having an ability to read lips under face masks. The framework employs Wi-Fi and radar technologies as enablers of RF sensing based Lip-reading. A dataset comprising of vowels A, E, I, O, U and empty (static/closed lips) is collected using both technologies, with a face mask. The collected data is used to train machine learning (ML) and deep learning (DL) models. A high classification accuracy of 95% is achieved on the Wi-Fi data utilising neural network (NN) models. Moreover, similar accuracy is achieved by VGG16 deep learning model on the collected radar-based dataset.

## Abstract (web summary)

This study aims to solve the fundamental limitations of camera-based systems by proposing a contactless and privacy-preserving radio frequency-based lip-reading framework, which can recognise lip movements under face masks.

## Introduction

Normal hearing is defined as the ability to hear a sound of 20 dB level and above. Inability to understand sounds of 20 dB and above can be recognised as hearing loss^1^. Hearing loss can be mild or severe and the subjects are referred to as ‘hard of hearing’. Hearing loss and deafness are a major impediment to normal communication and learning. Overall, 5% of the world’s population, around 430 Million people, suffer from hearing impairments. The number is expected to increase to 700 million people by 2050^1^. In the United Kingdom (UK) alone, around 11 million individuals live with hearing impairments and age-related hearing loss has become a serious concern^2^.

**Fig. 1:**
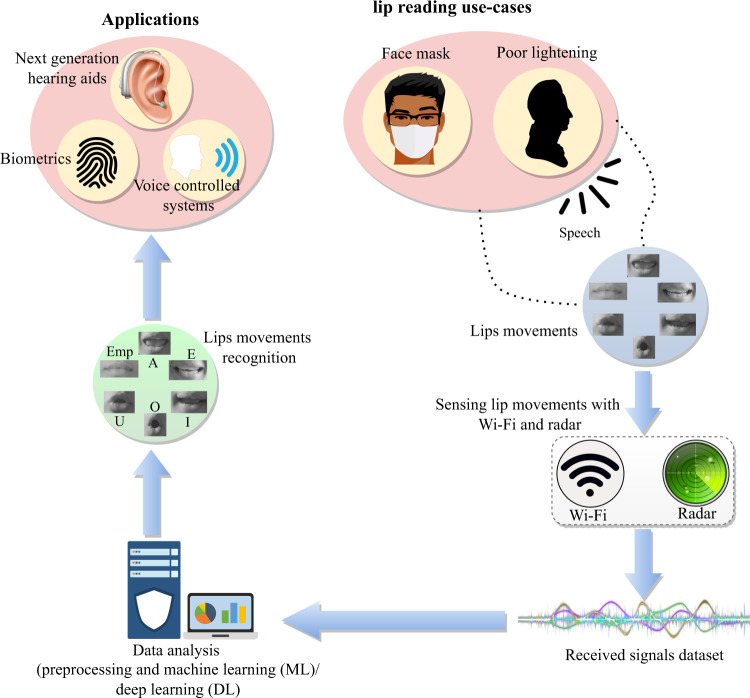
Conceptual illustration of the proposed Lip-reading framework. The framework employs Wi-Fi and radar technologies as enablers of RF sensing based Lip-reading. A dataset comprising of vowels A, E, I, O, U and empty (static/closed lips) is collected using both technologies, with a face mask. The collected data is used to train ML and DL models.

Next-generation hearing aids by 2050 require transformative multi-modal processing, uninhibited by limitations of speech or sound enhancement. We humans use also visual information for the cognition of spoken words and not just limited to sound alone. Visual information, such as Lip reading is an important aspect of speech recognition. Unfortunately, visual information obtained for hearing aids through cameras suffer from privacy issues. The legal ramifications of such aids alone may inhibit their widespread use in public and private spaces, e.g. video in hearing aids may be considered as filming someone without consent, which is illegal in many parts of the world. These days, hearing aids assisted by visual information have suffered from restrictions chief among them is the face mask in the coronavirus (COVID-19) era. The demand for next-generation hearing aids can be fulfilled by radio frequency (RF) sensing of lip and mouth movements. Lip reading through RF sensing can provide highly accurate cues to the hearing aids by identifying spoken sounds and detecting speech patterns through machine learning (ML) and deep learning (DL) techniques. Furthermore, unlike vision-based systems, RF-sensing-based Lip reading does not suffer from limitations due to face masks. RF signals can penetrate the mask to capture visual cues including lip and mouth movements, which will otherwise be obscured from visual hearing aids. This provides an exciting opportunity to transform next-generation multi-modal hearing aids through RF sensing. The system may just require the addition of a single antenna on the hearing aid. In this work, we have designed, developed and demonstrated a working RF sensing-based solution for detecting spoken sounds through face masks. The proposed RF sensing system can either work as a standalone system or assist in sensing for hearing aids through reading of lip and mouth movements in the presence of face masks, which normally obstruct visual cues for hearing aids in vision-based systems. A conceptual illustration of the proposed Lip-reading framework is presented in Fig. 1.

The lip and mouth movements result in variations in the wireless channel state information (CSI) amplitudes, which are picked up by ML/DL algorithms as patterns belonging to spoken sounds and classified into their respective speech, words, phonemes or spoken letters. For completeness, both RF-based sensing systems, i.e., Wi-Fi and radar have been demonstrated. The radar-based system utilises Doppler shift spectrograms, which are identified by a DL model to classify different lip movements. The proposed lip-reading framework has a potential value in many applications, including hearing aid devices, biometric security, and voice-enabled control systems in smart homes and cars infotainment.

Evidently, Lip reading has gained notable research attention in recent years due to its significance in many applications, such as communicating with deaf community and biometric authentication^3^ to identifying individuals based on the visual information obtained from lip movements. In this regard, a Lip-reading-based surveillance system has been proposed in^4^. Moreover, Lip reading has been studied in the area of audio-video speech enhancement (AVSE) to enhance speech with the aid of visual information collected from the camera^5^. Similarly, visual speech recognition is another area wherein only visual features are used to perform Lip reading without the aid of any audio data^6^. A Lip-reading framework to predict Greek phrases using a mobile phone’s frontal camera with convolutional neural networks (CNN) and temporal neural networks (TCN) architecture is proposed in ref. 7.

However, all camera-based systems have certain fundamental flaws, such as the obligation to record the target, which limits the real-world applications due to privacy concerns. Moreover, poor lighting also has an impact on the quality of recorded images/videos. Lip reading in the presence of face masks with camera-based technologies has become potentially impossible in the COVID-19 era. Camera-based Lip-reading systems also fail in complete darkness when lip movements cannot be visually observed. This paper aims at resolving the aforementioned limitations via RF sensing which revives the above-mentioned applications in COVID-19 era, where face covering has become a norm.

Recently, RF sensing has been considered in the context of assisted living, and contactless monitoring of activities (i.e., end-users do not need to wear or carry devices, or change their daily routine) along with the possibility of leveraging existing communication signals and infrastructure, such as common Wi-Fi routers. RF signals are being used to monitor both macro and micro movements^8–11^. Witrack^12^ developed a 10cm granularity frequency-modulated continuous wave (FMCW) 3D motion tracking system. Similarly, WISee^13^ used Doppler shifts to detect gestures. Moreover, Allsee^14^ utilised custom RFID tags to achieve low-power gesture recognition. Device-free RF-based human localisation systems have been used to determine a person’s position by measuring one’s impact on wireless signal variations, received by pre-deployed monitors^15^. The authors in ref. 16 produced a FMCW radar system to determine the Doppler, temporal changes, and radar cross sections of falling and other fall-related activities. The authors demonstrated that wireless waves through the use of radar systems can be used to classify human motion. The work in ref. 17 utilised CSI of Wi-Fi orthogonal frequency-division multiplexing (OFDM) signals for the classification of five different arm movements. The subjects made different arm gestures, while standing between a Wi-Fi router and a laptop that were both transmitting wireless signals. The CSI was recorded, and the gestures were classified using ML algorithms. In ref. 18, the authors detected multiple user activities based on variations in CSI of wireless signals using deep learning networks. The work in ref. 19 collected a dataset for sitting and standing movements, utilising RF signals obtained from software defined radios (SDR). Patterns in the wireless signals capture different body motions, as each movement induces a unique change in the wireless medium. In ref. 20, human movements are detected in real time via universal software radio peripheral (USRP) devices to form a wireless communication link where signal propagation data is recorded when a user moves or remains motionless and machine learning is used. The work in ref. 21 provided a speech recovery technique based on a 24-GHz portable auditory radar and a webcam for speech recognition. Different subjects only speak a single English letter “A”.

Wang et al.^22^ developed a CSI-based recognition system that can identify what people are saying. The presented system functions similar to Lip reading and utilised CSI signals to detect mouth movements during speech. By employing beam forming, the signal was directed towards the user’s mouth. The mouth motions were then decoded in two stages. The initial step was to filter out interference before using discrete wavelet packet decomposition to create mouth movement profiles. The profiles were then classified with ML techniques to determine pronunciations. This research shows how Wi-Fi may be used to detect lip movements. However, COVID-19 pandemic has introduced many limitations, such as face masks and RF sensing systems have not been demonstrated.

A related work is presented in ref. 23, where authors use the structural principle and electrical properties of the flexible triboelectric sensors to decode the lip movements. The sensors are placed inside a pseudo mask where the lips remained exposed to make them clearly visible. Although the authors achieve promising results in identifying lip movements, their solution cannot be generalised to realistic situations and suffers from limitations of wearing sensors and exposing lips in the COVID-19 era.

Herein, we have utilised RF sensing to detect lip movements remotely and extract motion information from the mouth movements during speech, in the presence of face masks. The motivation behind utilising both RF sensing techniques is to demonstrate the performance of both techniques to reveal which technique may perform better than the other in terms of accuracy. Our approach can transform multi-modal hearing aids under COVID-19 conditions and norms. Our proposed technique achieved high classification performance compared to the current state-of-the-art methods, such as vision-based systems and other hearing aid assistive technologies. We proposed different techniques and AI approaches to read lips using radar and Wi-Fi signals. Each scenario examined six different scenarios with spoken sounds for vowels, including lip/mouth movements and facial expressions: A, E, I, O, U, and Empty (Silence). Various ML and DL methods are examined using three male and female participants to correctly categorise the considered face postures with and without face mask.

## Results

**Fig. 2:**
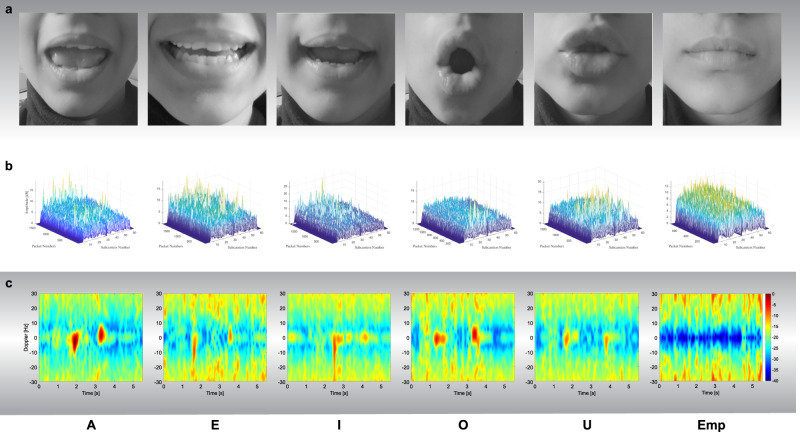
Pronounced vowels with their representation in Wi-Fi and radar signal. **a** A visual illustration of the pronounced vowels. **b** Wi-Fi data samples with mask representing various vowels classes. **c** Radar data samples with mask representing various vowels classes.

The conducted experiments were performed with two different technologies, i.e., Wi-Fi and radar. Five vowels, A, E, I, O, and U were collected with an empty letter, where subjects were not talking at all, and the lips were in normal closed position. An illustration of the lip movements to speak out all classes is shown in Fig. 2a, while the corresponding CSI samples and spectrograms are shown in Fig. 2b, c, respectively. In what follows, the experimental hardware setup to collect these data using both technologies is expressed.

For both experiments (radar and Wi-Fi), three participants, one male and two females, participated in the data collection process. The reason to include more participants was to make the dataset more realistic and diverse. A total of 3600 data samples were collected during both experiments for six classes, namely, A, E, I, O, U, and Emp, where Emp represents the lip posture of being silent. In each experiment, a total of 1800 data samples were collected from three participants, 900 with face mask and 900 without a face mask, where 50 samples were collected in each class. In particular, each participant repeated the speaking activity of each vowel 50 times with a mask and 50 times without a mask with the radar. Similarly, the same amount of data was collected from USRP with the same strategy. In this way, each participant contributed to collect 1200 data samples in total for six classes, two scenarios (with mask and without mask) and two technologies (radar and Wi-Fi). The ethical approval to conduct these experiments was obtained by the University of Glasgow’s Research Ethics Committee (approval no.: 300200232, 300190109).

### Radar-based setup

**Fig. 3:**
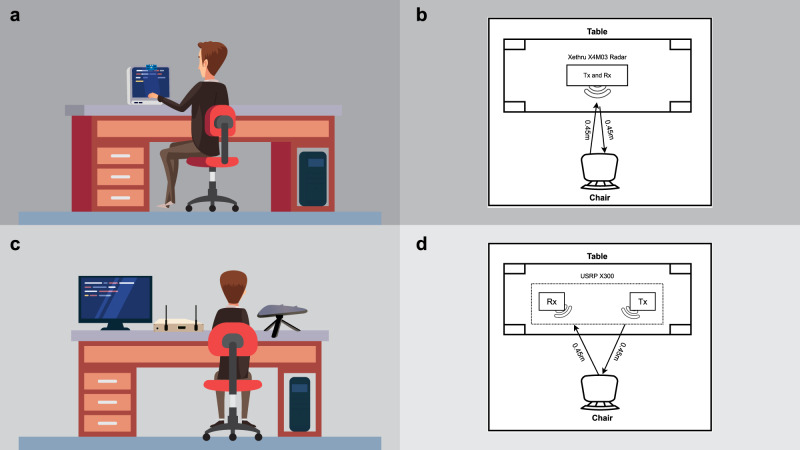
Experimental setup of the data collection through radar and Wi-Fi. **a** Front view of the data collection setup using Xethru UWB radar. **b** Top view of the radar-based data collection. **c** Front view of Wi-Fi based data collection. **d** Top view of the Wi-Fi-based data collection setup.

**Fig. 4:**
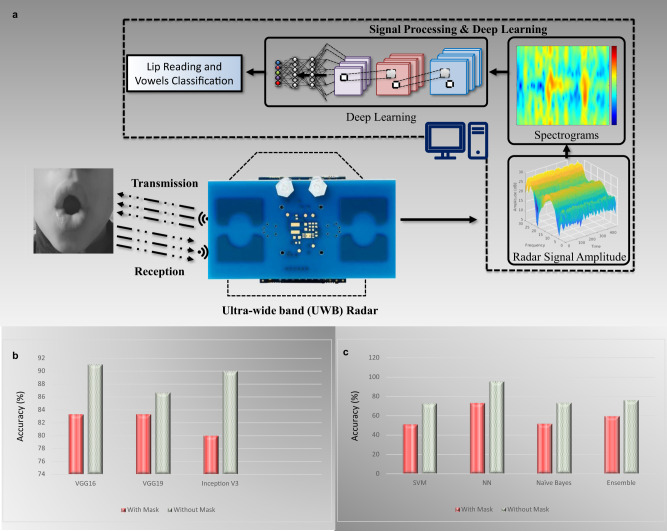
Overall system overview and the results bar graphs. **a** Radar-based system overview and data collection for Lip reading. **b** The accuracy improvement of male subject using DL algorithms between with mask and without mask using radar. **c** The accuracy improvement of Male subject using ML algorithms between with mask and without mask using Wi-Fi.

The hardware setup of radar-based Lip-reading system is shown in Fig. 3, where Fig. 3a shows the front view and Fig. 3b represents the top view. Correspondingly, the front and top views of Wi-Fi-based setup are shown in Fig. 3c, and d, respectively. For radar-based setup, an ultra-wide band (UWB) radar sensor, Xethru X4M03, was used in this experiment, which was placed on top of the screen of the laptop. The Xethru X4M03 is a UWB radar sensor with built-in transmitter (Tx) and receiver (Rx) antennas, providing a maximum detection range of 9.6 m. Key parameter settings of the radar are indicated in Table 1. The subject was sitting 0.45 m away from the radar while pronouncing vowels, as illustrated in 3b. The body was in a normal position and the only movements were the lip movements along with slight head movements, which are common while talking. The duration of each activity was set to 6 seconds, where an activity represents the data collection of a single vowel from a single subject. The RF signal was transmitted and received from the radar within this duration. The UWB radar-based system setup for Lip-reading data collection and processing is illustrated in Fig. 4a. The details of all components presented in the figure are discussed later in this section. The features utilised for the radar are obtained from the short time Fourier transform (STFT) of the radar signal which provides the spectrograms of radar Doppler shift due to lip and mouth movements. The analysis of the spectrograms showed that different vowels resulted in different spectrograms due to the differences in lip and mouth movements. To classify vowels, pre-trained VGG models were utilised due to their better performance on abstract images like spectrograms^24,25^.

**Table 1:** Configuration parameters of radar software and hardware

Parameter	Value
Platform	Xetru radar X4MO3
Instrumental range	9.6 m
Target’s distance from radar	0.45 m
Operating frequency	7.29 GHz
Transmitter power	6.3 dBm
Activity duration	6 s
Collected samples in each class	50

### Wi-Fi-based setup

For the second set of experiments, Wi-Fi was used as a lip movement recognition platform. For this, a USRP X300 was used, equipped with one directional antenna as a transmitter (Tx) and two omnidirectional antennas as receivers (Rx), as shown in Fig. 3c. For experiments, monopole antennas, VERT2450, optimised at 2.45 GHz frequency band, were used as Rx. A log-periodic antenna, HyperLOG 7040 X BPA, was used as a Tx. Both Tx and Rx antenna gains were set to 35 dB. The USRP was connected with a desktop having an Intel(R) Core (TM) i7-7700 3.60 GHz processors with 16GB RAM. Key parameter settings of the Wi-Fi-based setup are indicated in Table 2. GNU radio was used to communicate with the USRP with the help of a virtual machine having Ubuntu 16.04 operating system. A python script was developed to send and receive data from USRP X300. The experiments were conducted at an operational frequency of Wi-Fi in 2.45 GHz band. Both the Tx and Rx antennas were placed around 0.45 m from the target, as illustrated in Fig. 3d. Each activity was performed for 6 s. The Wi-Fi-based system setup for Lip-reading data collection and processing is presented in Supplementary Fig. 1 and details of all components presented in the figure are discussed later in this section. It is worth mentioning that Wi-Fi signals were tested with different features including time–frequency maps, etc. However, the CSI values of Wi-Fi signals performed best with variations in CSI amplitudes unlike the radar signals, where frequency shift was a major differentiating factor. The variations in one-dimensional CSI amplitude showed clear patterns which could be attributed to a spoken vowel.

**Table 2:** Configuration parameters of USRP software and hardware

Parameter	Value
USRP Platform	X300
OFDM subcarriers	51
Operating frequency	2.45 GHz
Transmitter Gain	35 dB
Receiver gain	35 dB
TX Antenna	Log periodic HyperLOG 7040, 700 MHz to 4 GHz
Rx Antenna	Monopole VERT2450, 2.45 GHz
Target’s distance from Tx and Rx antennas	0.45 m
Activity duration	6 s
Collected samples in each class	50

In what follows, the performance of considered ML and DL algorithms on the datasets collected from Wi-Fi and radar for the cases of with face mask and without face mask is discussed.

### Evaluation metrics of classification models

The performance of the DL and ML models in the classification of vowels is evaluated through accuracy, true positive Rate (TPR) and false Positive Rate (FPR). TPR and FPR are calculated using Eqs. (1) and (2), respectively. Further, accuracy, which is one of the most commonly used metrics in the literature for classification, is calculated using Eq. (3).

1 $${{{{{\rm{TPR}}}}}}=\frac{{{{{{\rm{TP}}}}}}}{{{{{{\rm{TP}}}}}}+{{{{{\rm{FN}}}}}}}$$

2 $${{{{{\rm{FPR}}}}}}=\frac{{{{{{\rm{FP}}}}}}}{{{{{{\rm{FP}}}}}}+{{{{{\rm{TN}}}}}}}$$

3 $${{{{{\rm{Accuracy}}}}}}=\frac{({{{{{\rm{TP}}}}}}+{{{{{\rm{TN}}}}}})}{({{{{{\rm{TP}}}}}}+{{{{{\rm{FP}}}}}}+{{{{{\rm{TN}}}}}}+{{{{{\rm{FN}}}}}})}$$

where TP stands for true positive, i.e., both the truth and the predicted values are positive. FN is false negative, which represents the cases when the truth is positive and the prediction is negative.

### Radar data

The evaluation results of the considered DL algorithms (VGG16, VGG19, and InceptionV3) on the radar dataset are presented in Table 3. VGG and Inception are CNN-based deep learning models (trained on ImageNet dataset^26^), which are commonly used in image classification. VGG16, VGG19 and InceptionV3 have 16, 19 and deep layers, respectively. A detailed description of these models is presented in refs. 27, 28. Moreover, ref. 29 provides the fundamental understanding of ML.

**Table 3:** Comparative result of vowels with and without mask using radar

Subject	VGG16		VGG19		InceptionV3	
	With mask	Without mask	With mask	Without mask	With mask	Without mask
S1 (Male)	83.33%	91.07%	83.33%	86.67%	80.00%	90.00%
S2 (Female)	85.00%	83.03%	75.00%	81.67%	70.00%	80.00%
S3 (Female)	76.07%	85.00%	75.00%	76.07%	75.00%	80.00%
Combined	73.44%	85.94%	68.09%	79.69%	65.00%	73.44%

It can be observed from Table 3 that all algorithms produce comparable results with VGG16 slightly outperforming others on all individual subjects and combined datasets in terms of accuracy. Using VGG16, the classification accuracy of 91.7% is observed on S1 dataset without mask, which is reduced to a promising accuracy of 83.3% when the subject wears the face mask. The other performance metrics, such as TPR and FPR are presented in Supplementary Table 2 and 3. It can be observed from the tables that they perform well on all individual classes. Almost all individual classes produce 100% TPR with mask and promising TPR on without mask dataset. Similarly, on combined dataset the same algorithm produces best results in terms of classification accuracy for both with mask and without mask. Overall, a classification accuracy of 85.94% is observed without face mask on the combined dataset. On the other hand, the same algorithm classifies the vowels with 73.44% accuracy with a face mask on the combined dataset. Moreover, other DL models, i.e., VGG19 and Inception V3 also produce comparable results on the radar dataset.

Figure 4 b shows the accuracies of with mask and without mask scenarios for different DL algorithms examined on the radar data of the male subject. It can be observed from the figure that InceptionV3 produces biggest accuracy difference between with mask and without mask cases, which is around 12%, while VGG19 produces the least different, which is around just 4%. Overall, VGG16 performs better on both datasets with an accuracy difference of 7%.

#### Wi-Fi data

Table 4 represents the average accuracy of classifying the dataset collected from Wi-Fi using different ML and DL algorithms. Four different algorithms are considered, namely NN, support vector machine (SVM), Ensemble and Naïve Bayes. The results are generated using test-train split evaluation method. It can be noted from the tables that the NN algorithm outperforms others for individual male and female data and the combined dataset. Using NN algorithm, the classification accuracy of 95.6% is observed on S1 without face mask, while the same algorithm gives 73.3% classification accuracy on the same subject when he wears a face mask. Similarly, on the combined dataset, NN gives a premising accuracy of 73.3% without a face mask and an accuracy of 61.1% on the with-mask combined dataset. The other performance metrics, such as TPR and FPR are shown in Supplementary Tables 4 and 5. It can be observed that these metrics perform well on all individual classes. Almost all individual classes produce 100% TPR with mask and promising TPR on without mask dataset. Interestingly, the classification accuracy of male dataset for all algorithms is higher than the females’ dataset. This is due to the reason that the lip movements of male subject in pronouncing vowels were comparatively larger than the females among the participants.

**Table 4:** Comparison between different ML and DL algorithms in classifying vowels on the Wi-Fi dataset

Subject	Neural network Pattern recognition		SVM (Medium Gaussian SVM)		Ensemble (boosted trees)		Naïve Bayes (Kernel Naïve Bayes)	
	With mask	Without mask	With mask	Without mask	With mask	Without mask	With mask	Without mask
S1 (Male)	73.03%	95.06%	51.03%	73.00%	59.07%	76.03%	52.00%	73.03%
S2 (Female)	80.00%	76.03%	61.07%	65.00%	59.07%	61.03%	60.07%	62.07%
S3 (Female)	76.07%	88.09%	54.07%	61.07%	56.00%	62.07%	54.07%	62.03%
Combined	61.01%	73.03%	50.09%	57.08%	56.09%	57.08%	49.00%	57.08%

Overall, the classification accuracy of with mask dataset is lower than without the face mask. This is because of the reason that lip movements are restricted due to the restraints caused by the face mask. For instance, a person may not be able to fully open the mouth while wearing a face mask. The percentage accuracy difference in classifying with mask and without mask dataset is depicted in Fig. 4c. The biggest accuracy difference is observed for male subjects for NN algorithm where an accuracy difference of around 23%. The minimum difference observed is for ensemble algorithm on S1 dataset, where with mask and without mask accuracy difference is 12%.

## Discussion

In this study, a Lip-reading RF-sensing-based framework is proposed using both RF sensing technologies, i.e., Wi-Fi and radar. Wi-Fi signals are generated using USRP x300, which uses CSI signals to identify human lip movements for all considered classes. For radar, a UWB radar sensor, Xethru X4M03 was used, where reflected Doppler signals (Hz) were plotted in the form of frequency–time diagrams, such as spectrograms. The proposed RF sensing system can either work as a standalone system or assist in sensing for hearing aids through reading of lip and mouth movements in the presence of face masks, which normally obstruct visual cues for hearing aids in vision-based systems. A diverse dataset of three participants (one male and two females) was collected for 5 vowels A, E, I, O, U, and Empty, where lips were not moving. The collected dataset was used to train different ML and DL algorithms. The paper’s major goal was to propose a secure Lip-reading system that could identify the lip movements in the presence of a mask with different RF sensing technologies and ML/DL algorithms. In particular, four algorithms, NN, SVM, Ensemble, and Naïve Bayes, were evaluated using train-test evaluation methods on the Wi-Fi dataset, where the maximum classification accuracy of % was observed on the male datasets without a face mask. On the other hand, DL pre-trained models VGG16, VGG19, and InceptionV3 were evaluated using test-train methods and found a maximum average accuracy of 91.07% on male data without mask using radar. Moreover, because the current system is a proof of concept with the goal of showing the importance and effectiveness of detecting lips using RF-sensing technology such as radar and Wi-Fi, future experiments will be conducted to detect different words or sentences in real time and perform activity from various angles using radar and Wi-Fi. Furthermore, and as mentioned earlier, the dataset used to achieve the previously reported results is made publicly available to encourage other researchers and the wider communities to take this system a step further.

## Methods

In the case of Wi-Fi, each instance of the data represents the CSI amplitudes, where 2000 packets were transmitted in a duration of six seconds. Figure 2b illustrates the CSI patterns (amplitude) of considered lip movements, i.e., A, E, I, O, U and empty, in the case of face mask. The CSI patterns in the case of without face mask are illustrated in Supplementary Fig. 2. Different colours in each figure represent the 51 subcarriers of the OFDM signal. The *Y*-axis of each sub-figure represents the amplitude of the subcarriers while number of received packets are displayed on x-axis. The same data collection strategy was applied in radar, where a total number 1800 data samples were collected for three subject male and females with and without a face mask, with 50 data samples in each class. In the case of radar, each instance of data sample is represented in the form of a spectrogram, displayed in Fig. 2c for with face mask. The spectrograms for without face mask scenario are represented in Supplementary Fig. 3. Different colours in each figure represent change in frequency. The *Y*-axis of each sub-figure represents the Doppler (Hz) while time is displayed on the *x*-axis.

### Processing radar data

In the beginning, the radar chip was configured via the XEP interface with x4driver. Data were recorded from the module at 500 frames per second (FPS) in the form of the float message data. A loop was used to read the data file and save the data into a DataStream variable, which was mapped into a complex range-time-intensity matrix. Thereafter, moving target indication (MTI) filter was applied to get the Doppler range map. Afterwards, the second MTI was used as a Butterworth 4th order filter to generate the Spectrograms using the following parameters: window length, overlap percentage, and fast Fourier transform (FFT) padding factor. In particular, a window length of 128 samples, and a padding factor of 16 was used. In addition, a range profile was created by first converting each chirp to an FFT. A second FFT is then conducted on a defined number of consecutive chirps for a given range bin. Furthermore, an STFT was used to create these spectrograms, because, unlike Fourier transform, it offers both temporal and frequency information^30^. This is done by segmenting the data and then performing Fourier transform on each segment. When the window length is changed, both the temporal and frequency resolutions are altered inversely. For example, if one increases the other decreases. The level of Doppler detail in RADAR data is determined by the hardware’s sampling capability. The greatest unambiguous Doppler frequency in RADAR is ${F}_{d},\max=\frac{1}{2}{t}_{r}$, where *t*_*r*_ is the chirp time. In this paper, we look at Lip-reading recognition at a distance D(t) from a specified location such as the mouth. V(t) represents the point of target movement in front of the RADAR, and *T*_*s*_ represents the transmitted signal,

4 $${T}_{s}(t)=A\cos (2\pi ft).$$

The received signal is provided by Rs(t),

5 $${R}_{s}(t)={\acute{A}}\cos \left(2\pi f\left(t-\frac{2D(t)}{c}\right)\right),$$

where *A* is the reflection coefficient, and *c* is the speed of light. The reflected signal can be expressed as *R*_*s*_(*t*), where the signal reflected off the target points at an angle *θ* to the direction of RADAR.

6 $${R}_{s}(t)={\acute{A}}\cos \left(2\pi f\left(1+\frac{2v(t)}{c}\right)\left(t-\frac{4\pi D(\theta )}{c}\right)\right).$$

The Doppler shift that corresponds to it can be written as,

7 $${f}_{d}=f\frac{2v(t)}{c}.$$

The returned signal becomes a composite of several moving elements such as the head and lips. Each component moves at its own speed and acceleration. If we consider *i* to be the various moving components of the lip, we can write the received signal as

8 $${R}_{s}(t)=\mathop{\sum }\limits_{i}^{N}{A}_{i}\cos \left(2\pi f\left(1+\frac{2vi(t)}{c}\right)\left(t-\frac{4\pi {D}_{i}(0)}{c}\right)\right).$$

The Doppler shift is the result of a complex interaction of numerous Doppler shifts induced by different moving face parts. Detection of Lip reading in a reliable fashion clearly depends upon the characteristics of the Doppler signatures. After obtaining the spectrograms of various vowels and empty files from the participants a dataset was constructed. As indicated in the high-level signal flow diagram in Fig. 4a, the dataset consisted of two key modules: (i) System Training and (ii) System Testing. The proposed pre-trained DL classification algorithms were implemented on spectrogram to recognise vowels and the Empty dataset.

### Processing Wi-Fi data

The data were transmitted in the form of OFDM symbols comprising 52 closely spaced subcarriers. Data were collected in the form of a matrix that contains frequency responses of all *N* = 51 subcarriers, as shown in Eq. (9).

9 $$H={[{H}_{1}(\;f),{H}_{2}(f),\cdots,{H}_{N}(\;f)]}^{T},$$

Here frequency of each subcarrier *H*_*j*_ can be represented as

10 $${H}_{j}\left(f\right)=\vert {H}_{j}\left(f\right)|{e}^{\,j\angle {H}_{j}(\;f)},$$

where ∣*H*_*j*_(*f*)∣ and *∠H*_*j*_(*f*) are the amplitude and phase responses of the *j*th subcarrier. Each of these subcarrier responses is related to the system input and output as given in Eq. (11),

11 $${H}_{j}(f)=\frac{{Y}_{j}(\;f)}{{X}_{j}(\;f)},$$

where *Xj*(*f*) and *Yj*(*f*) are the Fourier transforms of input and output of the system. Indeed, the received CSI samples are impaired due to environmental noise. As a result, the collected samples are denoised by subtracting the mean received power from each subcarrier. To observe the maximum variation due to lip movements the subcarrier with highest variance was identified for the feature extraction. A total of 15 features were extracted namely, mean, median, standard deviation, variance, minimum, eight peaks and high order moments, such as skewness and kurtosis. The extracted features were stored in a comma separated values (CSV) file, which is used to train different ML and DL algorithms discussed later in this section. Thereafter, training, testing and validation were performed using test-train split evaluation method to accurately classify the vowels and empty class.

### Parameter settings of the considered algorithms

The proposed classification methodology to distinguish Lip-reading activities is divided into two key stages: (i) system training and (ii) system testing. In the case of radar data, the DL pre-trained models VGG16, VGG19, and InceptionV3^31^ were used on the spectrogram images generated from the radar data. While ML algorithms neural network pattern recognition, support vector machine (SVM, medium gaussian SVM), Ensemble (boosted trees) and Naïve Bayes (kernel Naïve Bayes) were used on Wi-Fi data. The parameter settings of ML and DL model are shown in Supplementary Table 6.

**VGG16 model:** VGG16 has been used with 16 convolution layers and a rectified linear unit (ReLU) activation function, with kernel sizes of 3 × 3. Following each convolution layer, a max-pooling layer with all kernel sizes of 2 × 2 was added. The final layer worked as three fully connected layers (FC). The convolution layer and FC hold the weight of the training results, which allows them to determine the number of parameters. The architecture of VGG16 with parameter settings is shown in Supplementary Fig. 4.

**VGG19 model:** A 3 × 3 filter was used to capture image details, consisting of five stages of convolution layers, five pooling layers, and three fully connected layers. The depth of the convolution kernel in the VGG19 network has been raised from 64 to 512, allowing for improved image feature vector extraction. A pooling layer was applied after each stage of convolutional layers. Each pooling layer has the same size and step size, which is 2 × 2.

**InceptionV3 model:** A 48-layered InceptionV3 DL model was also applied on the dataset. Three convolution layers were added first, followed by a max-pooling layer, two more convolution layers, and another max-pooling layer. The spectrograms were sent to various convolutions, which convoluted the input images using various filters, stacked the extracted data, and sent it forward, and this process was repeated multiple times across the network, rather than manually adjusting the filter size for each layer.

**Neural network pattern recognition model:** Data were passed through two-layer feed-forward networks with sigmoid hidden neurons, SoftMax output neurons, and scaled conjugate gradient backpropagation. Meanwhile, weight and bias values are updated according to the scaled conjugate gradient method. Training, validation, and test sets of data were created. Network performance was measured using cross-entropy and miss-classification errors.

**SVM (Medium Gaussian SVM) model:** SVM was used for the classification of dataset by determining the optimum hyperplane for separating data points from one class to another. Training data, parameter values, prior probabilities, support vectors, and algorithmic implementation details were stored in trained SVM classifiers. The experimental data were modelled using a Gaussian kernel.

**Ensemble (boosted trees) model:** Ensemble classifiers combined the results of a number of low-quality learners into a single high-quality ensemble model. Boosting ensemble method was used on the dataset to regulate the depth of tree learners by specifying the maximum number of splits or branch points. The experimental setup achieved better accuracy with 0.1 learning rate.

**Naïve Bayes(Kernel Naïve Bayes) model:** Naïve Bayes classifier was used for Lip-reading classification, which is based on the Bayes theorem and assumes that predictors are conditionally independent in the given class. Specifically, a Gaussian Naïve Bayes kernel was used in this experiment.

## Supplementary information

Supplementary Information

## Data Availability

The lip-reading dataset generated during the current study is available in the University of Glasgow’s repository (Enlighten), and can be accessed here^32^.
