# Supplementary material for: Pushing the limits of remote RF sensing by reading lips under the face mask
Source: Nat Commun. 2022 Sep 7;13:5168. doi: 10.1038/s41467-022-32231-1 (PMC9452506; doi:10.1038/s41467-022-32231-1)
Supplement: Supplementary file 1 — Supplementary Information [file 41467_2022_32231_MOESM1_ESM.pdf]

Supplementary Information

Contactless Lip Reading Under Face Masks Using RF Sensing and Deep Learning

Hira Hameed[1], Muhammad Usman[1], Ahsen Tahir[1,2], Amir Hussain[3], Hasan Abbas[3], Tie Jun Cui[4], Muhammad Ali Imran[1], and Qammer H. Abbasi[1,*]

[1]University of Glasgow, James Watt School of Engineering, Glasgow, G12 8QQ, UK

[2]Department of Electrical Engineering, University of Engineering and Technology, Lahore, PK.

[3]School of computing, Edinburgh Napier University, Scotland, UK

[4]State Key Laboratory of Millimetre Waves, Southeast University, Nanjing, China

*qammer.abbasi@glasgow.ac.uk

Supplementary Information

Supplementary note 1: The dataset 3

Supplementary note 2: The performance of DL models on the radar data. 3

Supplementary note 3: The parameter settings of ML/DL algorithms. 5

Supplementary note 4: Wi-Fi based lip reading system. 6

Supplementary note 5: Radar and Wi-Fi data representation without face mask. 6

Supplementary note 6: VGG-16 architecture and block diagram. 7

Supplementary note 1: The dataset

Supplementary table 1: An overview of the data collected, number of subjects and the activities performed

Subjects	Experimental Dataset																								
	Radar												Wi-Fi												
	With Mask						Without Mask						With Mask						Without Mask						
	A	E	I	O	U	Emp	A	E	I	O	U	Emp	A	E	I	O	U	Emp	A	E	I	O	U	Emp	Total
Subject1(S1)	50	50	50	50	50	50	50	50	50	50	50	50	50	50	50	50	50	50	50	50	50	50	50	50	1200
Subject2(S2)	50	50	50	50	50	50	50	50	50	50	50	50	50	50	50	50	50	50	50	50	50	50	50	50	1200
Subject3(S3)	50	50	50	50	50	50	50	50	50	50	50	50	50	50	50	50	50	50	50	50	50	50	50	50	1200
Total	150	150	150	150	150	150	150	150	150	150	150	150	150	150	150	150	150	150	150	150	150	150	150	150	
	900						900						900						900	900					3600

Supplementary note 2: The performance of DL models on the radar data.

Supplementary table 2: Comparative result of vowels with and without mask using radar dataset of subject 1 and 2.

DL Model		TPR/FPR (%)	S1(Male)							S1(Female)						
			A	E	I	O	U	Emp	Accuracy (%)	A	E	I	O	U	Emp	Accuracy (%)
VGG16	With Mask	TPR (%)	90.0	50.0	80.0	90.0	90.0	100	**83.3**	70.0	50.0	100	90.0	100	100	**85.0**
		FPR (%)	10.0	50.0	20.0	10.0	10.0	0.0		30.0	50.0	0.0	10.0	0.0	0.0	
	Without Mask	TPR (%)	80.0	100	80.0	90.0	100	100	**91.7**	86.7	63.3	83.3	83.3	83.3	100	**83.3**
		FPR (%)	20.0	0.0	20.0	10.0	0.0	0.0		13.3	36.7	16.7	16.7	16.7	0.0	
VGG19	With Mask	TPR (%)	70.0	80.0	90.0	80.0	80.0	100	**83.33**	50.0	45.0	92.0	90.0	82.0	90.0	**75.0**
		FPR (%)	30.0	20.0	10.0	20.0	20.0	0.0		50.0	55.0	8.0	10.0	18.0	10.0	
	Without Mask	TPR (%)	80.0	90.0	70.0	90.0	90.0	100	**86.67**	90.0	70.0	70.0	100	60.0	100	**81.67**
		FPR (%)	20.0	10.0	30.0	10.0	10.0	0.0		10.0	30.0	30.0	0.0	40.0	0.0	
InceptionV3	With Mask	TPR (%)	80.0	70.0	90.0	50.0	90.0	100	**80.0**	100	60.0	60.0	30.0	70.0	100	**70.0**
		FPR (%)	20.0	30.0	10.0	50.0	10.0	0.0		0.0	40.0	40.0	70.0	30.0	0.0	
	Without Mask	TPR (%)	100	90.0	50.0	100	100	100	**90.0**	80.0	70.0	90.0	50.0	90.0	100	**80.0**
		FPR (%)	0.0	10.0	50.0	0.0	0.0	0.0		10.0	30.0	10.0	50.0	10.0	0.0	

Supplementary table 3: Comparative result of vowels with and without mask using radar dataset of subject 3 and all combined.

DL Model		TPR/FPR (%)	S3(Female)							Combined						
			A	E	I	O	U	Emp	Accuracy (%)	A	E	I	O	U	Emp	Accuracy (%)
VGG16	With Mask	TPR (%)	50.0	80.0	100	70.0	60.0	100	**76.7**	75.0	67.0	44.0	67.0	67.0	100	**73.44**
		FPR (%)	50.0	20.0	0.0	30.0	40.0	0.0		25.0	33.0	56.0	33.0	33.0	0.0	

	Without Mask	TPR (%)	80.0	90.0	80.0	80.0	80.0	100	**85.0**	91.0	82.0	100	90.0	62.0	100	**85.94**
		FPR (%)	20.0	10.0	20.0	20.0	20.0	0.0		9.0	18.0	0.0	10.0	38.0	0.0	
VGG19	With Mask	TPR (%)	80.0	40.0	80.0	80.0	100	70.0	**75.0**	40.0	69.2	100	83.3	40.0	100	**68.9**
		FPR (%)	20.0	60.0	20.0	20.0	0.0	30.0		60.0	30.8	0.0	16.7	60.0	0.0	
	Without Mask	TPR (%)	50.0	80.0	100	70.0	60.0	100	**76.7**	88.0	73.0	100	36.0	78.0	100	**79.69**
		FPR (%)	50.0	20.0	0.0	30.0	40.0	0.0		12.0	27.0	0.0	64.0	22.0	0.0	
InceptionV3	With Mask	TPR (%)	80.0	40.0	80.0	80.0	100	70.0	**75.0**	76.0	60.0	36.0	46.0	82.0	90.0	**65.0**
		FPR (%)	20.0	60.0	20.0	20.0	0.0	30.0		24.0	40.0	64.0	54.0	18.0	10.0	
	Without Mask	TPR (%)	80.0	30.0	100	80.0	90.0	100	**80.0**	75.0	67.0	80.0	80.0	22.0	100	**73.44**
		FPR (%)	20.0	70.0	0.0	20.0	10.0	0.0		25.0	33.0	20.0	20.0	78.0	0.0	

Supplementary table 4: Comparative result of vowels with and without mask using Wi-Fi dataset of subject 1 and 2.

ML Model		TPR/FPR (%)	S1(Male)							S1(Female)						
			A	E	I	O	U	Emp	Accuracy (%)	A	E	I	O	U	Emp	Accuracy (%)
SVM (Medium Gaussian SVM)	With Mask	TPR (%)	54.0	40.0	42.0	38.0	46.0	88.0	**51.3**	38.0	72.0	58.0	70.0	36.0	96.0	**61.7**
		FPR (%)	46.0	60.0	58.0	62.0	54.0	12.0		62.0	28.0	42.0	30.0	64.0	4.0	
	Without Mask	TPR (%)	62.0	86.0	72.0	46.0	84.0	88.0	**73.0**	82.0	76.0	46.0	36.0	60.0	90.0	**65.0**
		FPR (%)	38.0	14.0	28.0	54.0	16.0	12.0		18.0	24.0	54.0	64.0	40.0	10.0	
Neural Network Pattern Recognition	With Mask	TPR (%)	60.0	100	60.0	60.0	75.0	100	**73.3**	50.0	75.0	71.4	85.7	50.0	100	**80.0**
		FPR (%)	40.0	0.0	40.0	40.0	25.0	0.0		50.0	25.0	28.6	14.3	50.0	0.0	
	Without Mask	TPR (%)	100	100	100	100	60.0	100	**95.6**	68.0	96.0	74.0	54.0	80.0	86.0	**76.3**
		FPR (%)	0.0	0.0	0.0	0.0	40.0	0.0		32.0	4.0	26.0	46.0	20.0	14.0	
Naive Bayes (Kernel Naive Bayes)	With Mask	TPR (%)	58.0	52.0	44.0	34.0	30.0	94.0	**52.0**	38.0	80.0	56.0	76.0	18.0	96.0	**60.7**
		FPR (%)	42.0	48.0	56.0	66.0	70.0	6.0		62.0	20.0	44.0	24.0	82.0	4.0	
	Without Mask	TPR (%)	60.0	88.0	78.0	40.0	76.0	98.0	**73.3**	76.0	66.0	58.0	22.0	58.0	96.0	**62.7**
		FPR (%)	40.0	12.0	22.0	60.0	24.0	20.0		24.0	34.0	42.0	78.0	42.0	4.0	
Ensemble (Boosted Trees)	With Mask	TPR (%)	58.0	80.0	42.0	66.0	16.0	96.0	**59.7**	58.0	80.0	42.0	66.0	16.0	96.0	**59.7**
		FPR (%)	42.0	20.0	58.0	34.0	84.0	4.0		42.0	20.0	58.0	34.0	84.0	4.0	
	Without Mask	TPR (%)	68.0	96.0	74.0	54.0	80.0	86.0	**76.3**	66.0	62.0	44.0	48.0	54.0	94.0	**61.3**
		FPR (%)	32.0	4.0	26.0	46.0	20.0	14.0		34.0	38.0	56.0	52.0	46.0	6.0	

Supplementary table 5: Comparative result of vowels with and without mask using Wi-Fi dataset of subject 3 and all combined.

ML Model		TPR/FPR (%)	S3(Female)							Combined						
			A	E	I	O	U	Emp	Accuracy (%)	A	E	I	O	U	Emp	Accuracy (%)
SVM (Medium Gaussian SVM)	With Mask	TPR (%)	76.0	10.0	62.0	60.0	24.0	96.0	**54.7**	32.7	46.0	36.7	56.0	34.0	100	**50.9**
		FPR (%)	24.0	90.0	38.0	40.0	76.0	4.0		67.3	54.0	63.3	44.0	66.0	0.0	
	Without Mask	TPR (%)	56.0	56.0	52.0	56.0	60.0	90.0	**61.7**	48.0	40.0	44.7	42.0	67.3	100	**57.8**
		FPR (%)	44.0	44.0	48.0	44.0	40.0	10.0		52.0	60.0	55.3	58.0	32.7	0.0	
Neural Network Pattern Recognition	With Mask	TPR (%)	60.0	88.0	78.0	40.0	76.0	98.0	**76.7**	41.7	50.0	61.5	63.2	35.7	100	**61.1**
		FPR (%)	40.0	12.0	22.0	60.0	24.0	2.0		58.3	50.0	38.5	36.8	64.3	0.0	
	Without Mask	TPR (%)	100	100	83.3	83.3	66.7	100	**88.9**	60.0	100	60.0	60.0	75.0	100	**73.3**
		FPR (%)	0.0	0.0	16.7	16.7	33.3	0.0		40.0	0.0	40.0	40.0	25.0	0.0	
Naive Bayes (Kernel Naive Bayes)	With Mask	TPR (%)	80.0	8.0	64.0	72.0	14.0	90.0	**54.7**	36.7	45.3	39.3	46.0	27.3	100	**49.0**
		FPR (%)	20.0	92.0	36.0	28.0	86.0	10.0		63.3	54.7	60.7	54.0	72.7	0.0	
	Without Mask	TPR (%)	58.0	58.0	38.0	58.0	64.0	98.0	**62.3**	34.7	46.7	62.0	25.3	48.7	95.3	**52.1**
		FPR (%)	42.0	42.0	62.0	42.0	36.0	2.0		65.3	53.3	38.0	74.7	51.3	4.7	
Ensemble (Boosted Trees)	With Mask	TPR (%)	64.0	50.0	40.0	40.0	46.0	96.0	**56.0**	44.0	56.0	40.7	63.3	37.3	100	**56.9**
		FPR (%)	36.0	50.0	60.0	60.0	54.0	4.0		56.0	44.0	59.3	36.7	62.7	0.0	
	Without Mask	TPR (%)	60.0	56.0	56.0	54.0	58.0	92.0	**62.7**	50.0	51.3	48.0	33.3	64.0	100	**57.8**
		FPR (%)	40.0	44.0	44.0	46.0	42.0	8.0		50.0	48.7	52.0	66.7	36.0	0.0	

Supplementary note 3: The parameter settings of ML/DL algorithms.

Supplementary table 6: Parameter settings for the selected models.

DL/ML Model	Parameters	Settings
VGG16	Number of layers	16
	Initial learning rate	0.0001
	Mini-batch size	16
	Learning algorithm	Adam
	Loss function	Cross entropy
	Maximum epochs	25
	Iteration per epoch	50
VGG19	Initial number of layers	19
	learning rate	0.0001
	Mini-batch size	16
	Learning algorithm	Adam
	Loss function	Cross entropy
	Maximum epochs	25
	Iteration per epoch	500
InceptionV3	Number of layers	48
	Initial learning rate	0.0001
	Mini-batch size	16
	Learning algorithm	Adam
	Loss function	Cross entropy
	Maximum epochs	25
	Iteration per epoch	500
NN	Initial number of layers	10
	Training Function	Scaled Conjugate Gradient Backpropagation
	Number of Epoch	20
	Loss function	Cross entropy
SVM	Kernel scale	3.9
	K-Fold predict	5
	Kernel function	Gaussian
	Loss function	Classifier
	Multiclass method	One-vs-One
Ensemble	Ensemble method	AdaBoost
	Learner type	Decision Tree
	Maximum number of splits	20
	Learning rate	0.1
	Number of learners	30
	Loss function	Classifier
Naïve Bayes	Kernel smooth density	Unbounded
	K-fold predict	5
	Kernel function	Gaussian
	Loss function	Classiferror
	Predictor distribution	mvmn

Supplementary note 4: Wi-Fi based lip reading system.

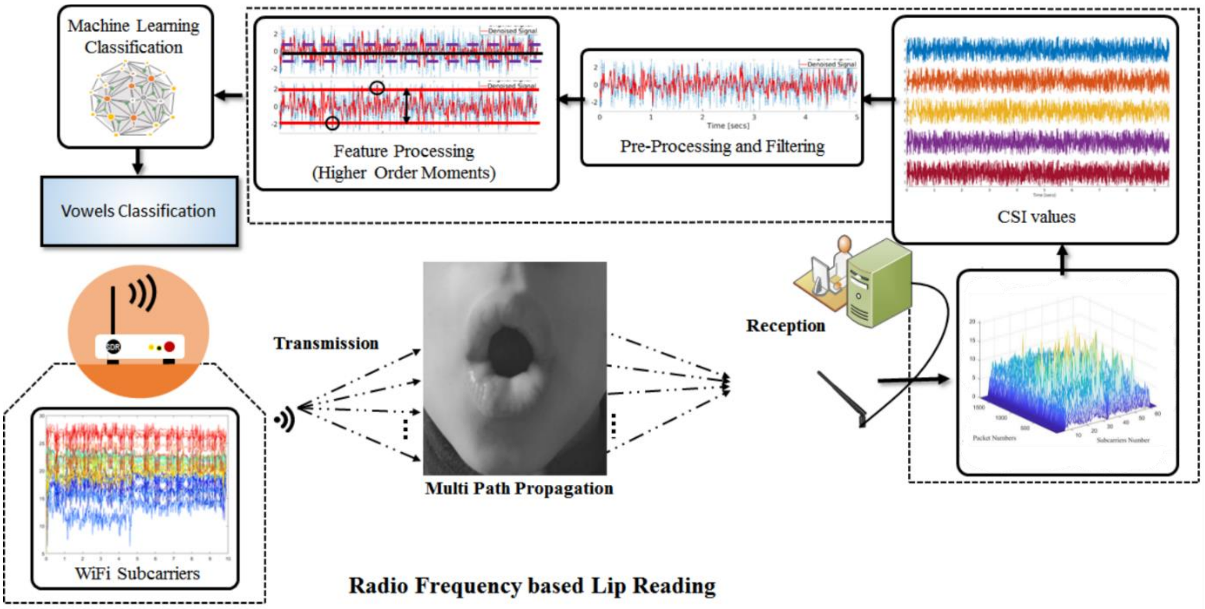

Supplementary Figure 1: Wi-Fi-based system overview and data collection for Lip-reading

Supplementary note 5: Radar and Wi-Fi data representation without face mask.

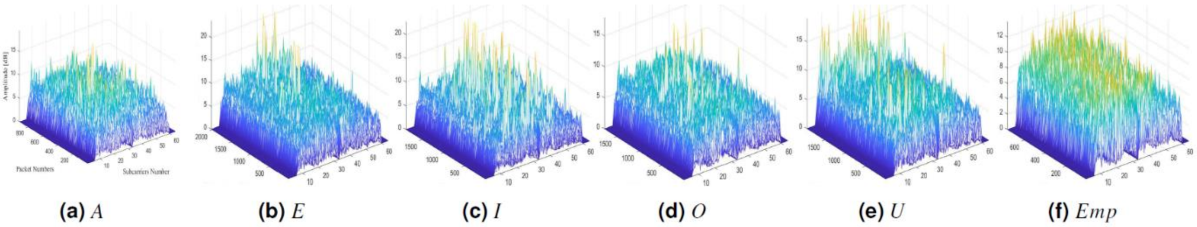

Supplementary Figure 2: Wi-Fi data samples without mask representing various vowels classes.

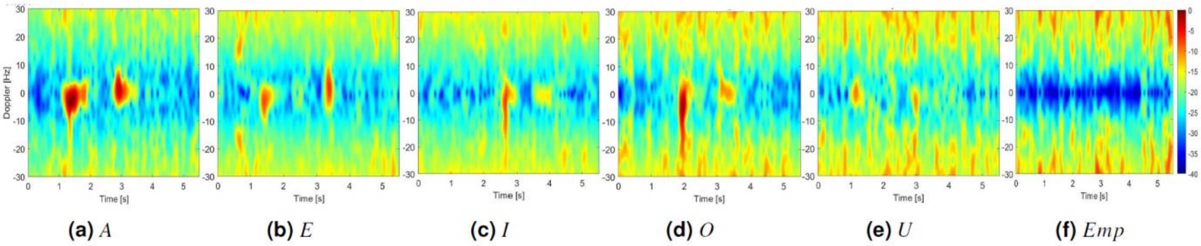

Supplementary Figure 3: Radar data samples without mask representing various vowels classes.

Supplementary note 6: VGG-16 architecture and block diagram.

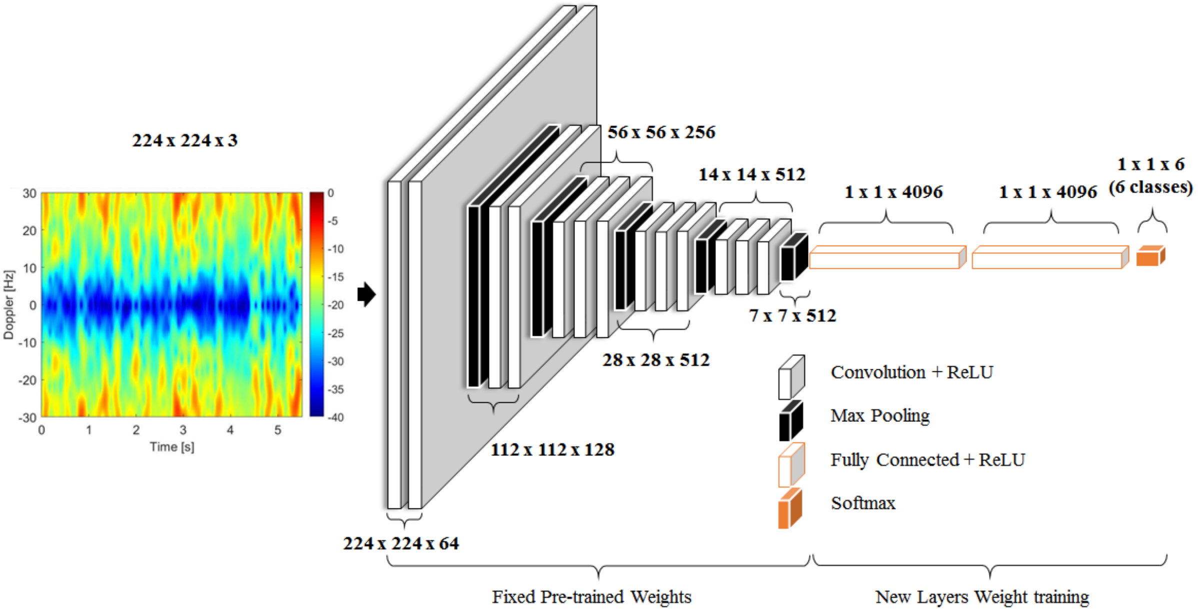

Supplementary Figure 4: VGG-16 with spectrogram input.

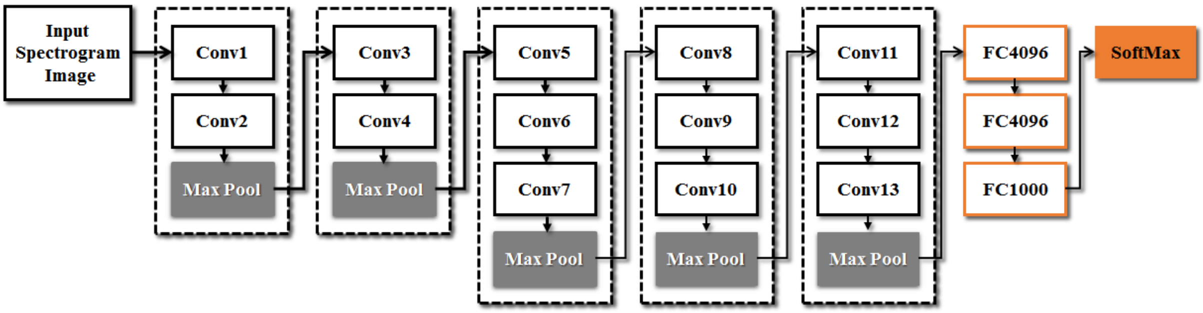

Supplementary Figure 5: VGG-16 block diagram.
